# Relationship between plasma TNF-α levels and agitation symptoms in first episode patients with schizophrenia

**DOI:** 10.1186/s12888-024-05796-y

**Published:** 2024-07-02

**Authors:** Xiaoying Wang, Wenjin Chen, Mengzhuang Gou, Wei Li, Na Li, Jinghui Tong, Yanfang Zhou, Ting Xie, Ting Yu, Wei Feng, Yanli Li, Song Chen, Baopeng Tian, Shuping Tan, Zhiren Wang, Shujuan Pan, Xingguang Luo, Ping Zhang, Junchao Huang, Li Tian, Chiang-Shan R. Li, Yunlong Tan

**Affiliations:** 1 Peking University HuiLongGuan Clinical Medical School, Beijing Huilongguan Hospital, Beijing, China; 2 Department of Psychiatry, Yale University School of Medicine, New Haven, CT USA; 3 Department of Physiology, Institute of Biomedicine and Translational Medicine, Faculty of Medicine, University of Tartu, Tartu, Estonia

**Keywords:** Schizophrenia, Agitation, TNF-α PANSS-EC

## Abstract

**Background:**

Increasing evidence suggested that immune abnormalities involved in the pathophysiology of schizophrenia. However, the relationship between immunity and clinical features has not been clarified. The aim of this study was to measure the plasma levels of tumor necrosis factor alpha (TNF-α) and soluble TNF-α receptor 1 (sTNF-α R1) and to investigate their association with agitation in first episode patients with schizophrenia (FEPS).

**Methods:**

The plasma TNF-α and sTNF-α R1 levels were measured using sandwich enzyme-linked immunosorbent assay (ELISA) in the FEPS with (*n* = 36) and without agitation (*n* = 49) symptoms, and healthy controls (HCs, *n* = 54). The psychopathology was assessed by the Positive and Negative Syndrome Scale (PANSS), and the agitation symptoms were evaluated by the PANSS excitatory component (PANSS-EC).

**Results:**

The plasma TNF-α levels in patients with and without agitation symptoms were significantly higher than those in HCs. The patients with agitation had significantly higher plasma TNF-α levels compared to the patients without agitation. There were no significant differences in the sTNF-α R1 levels among the three groups. Furthermore, the plasma TNF-α levels were positively correlated with the PANSS total score, Positive and General psychopathological subscores, and PANSS-EC score in the FEPS, but the relationships were not found for the plasma sTNF-α R1 levels.

**Conclusions:**

These results suggested that TNF-α might play an important role in the onset and development of agitation symptoms of schizophrenia.

## Introduction

Schizophrenia is characterized by positive symptoms, negative symptoms, and cognitive impairment [1]. Agitation is recognized as a component of the behavioral characteristics of schizophrenia. It can manifest as increased responsiveness to stimuli, irritability, excitement, physical or verbal aggression, and non-aggressive behaviors. Severely agitated patients represent a risk to themselves, their caregivers, and health-care providers [2]. Acute agitation is attributed to hallucinations, paranoid delusions, intense irritability, anger, and expansive mood, however, little is known about the pathophysiological mechanism of this state. Generally, abnormal increases in dopamine and noradrenaline (norepinephrine) and decreases in γ-aminobutyric acid (GABA) are considered underlying conditions for agitation [3].

Inflammatory immune markers are reportedly associated with the development of agitation symptoms in schizophrenia, and accumulating evidence shows immune system disturbances in severe mental disorders. Some studies showed that the IL-18 system might be part of the biological correlates of agitation, independently of affective and psychotic symptoms [4]. Inpatients with agitation presented a significantly higher C-reactive protein (CRP) compared to controls; after treatment, a significant reduction in CRP and excitement symptoms of Positive And Negative Syndrome Scale (PANSS) was observed [5]. Inpatients with elevated CRP displayed stronger aggressive behavior compared to patients with normal CRP levels (< 1 mg/dL). This difference manifested as higher rates of restraint during hospitalization and higher PANSS excitatory component (PANSS-EC) scores. Higher PANSS-EC scores were found to be associated with elevated CRP levels after controlling for the covariates of age, sex, BMI, and smoking [6].

Higher serum CRP and free triiodothyronine (FT3) levels and lower thyrotropin (TSH) levels were found to be independent risk factors for agitation in hospitalized patients with schizophrenia. In addition, inflammation abnormality may be involved in the pathogenesis of agitation in schizophrenia [7]. Some studies reported that increased tumor necrosis factor alpha (TNF-α) levels were correlated with the psychopathology in first-episode drug-naïve patients, which suggested that inflammatory cytokines might play a crucial role in the etiopathogenesis of schizophrenia [8]. TNF-α is the prototype of pro-inflammatory cytokine, binds to the TNF R1 and TNF R2 receptors. Two receptors are abundant in cell membrane of leucocytes and are soluble in serum. Circulating level of TNF receptors, especially sTNFR1, is a stable and reliable marker of the activity in the TNF-α system. The receptors are induced by TNF-α and measurement of their concentration is useful to determine the overall production of TNF-α [9].

In view of these broad neurobiological functions of inflammatory markers and cytokines, it has been suggested that they could affect the psychotic symptoms of schizophrenia, and also agitation. Several previous studies have provided limited encouraging evidence supporting this hypothesis. The aim of this study was to assess the clinical significance of the plasma levels of TNF-α and sTNF-α R1 in first episode patients with schizophrenia (FEPS), and their potential association with agitation.

## Methods

### Subjects

Eighty-five patients were recruited from inpatients at the Beijing HuiLongGuan Hospital. The ethical approval number is 2022-15 Research. The inclusion criteria were as follows: (1) diagnosis of schizophrenia confirmed by two psychiatrists based on the Structured Clinical Interview for DSM-IV (SCID); (2) age 15–55 years old; (3) previous antipsychotic exposure did not exceed 2 weeks [10, 11]; and (4) total disease duration ≤ 60 months. The exclusion criteria were the following: (1) other psychiatric disorders meeting DSM-IV Axis I diagnosis; (2) co-morbidity of acute, unstable, and/or significant untreated medical illnesses; (3) documented disease of the central nervous system (CNS); (4) history of chronic inflammation, autoimmune disorder, or severe allergy; (5) pregnancy or breastfeeding state; (6) dependence on alcohol or illicit drugs; (7) use of non-steroid anti-inflammatory drugs within the previous 4 weeks; and (8) no capacity to understand the study and provide written informed consent.

In addition, 54 sex-matched healthy volunteers were enrolled from local communities. The exclusion criteria were as follows: (1) alcohol and/or substance dependence; (2) previous diagnosis of a psychiatric disorder; (3) physical organ diseases, including thyroid, kidney, gout, brain, or immune diseases, diabetes, or other serious medical conditions; (4) infection in the previous 4 weeks or current use of anti-inflammatory drugs, glucocorticoids, or antibiotics; (5) pregnancy or breastfeeding; and (6) family history of psychiatric disorders. The study was approved by the Institutional Review Board (IRB) of the Beijing Huilongguan Hospital, and all subjects provided written informed consent to participate. The demographic data for patients and control subjects are shown in Table 1.

**Table 1:** Demographics, clinical characteristics and TNF-α and sTNF-α R1 levels of paticipants (mean ± SD)

	Schizophrenics without agitation (*N* = 49)	Schizophrenics with agitation (*N* = 36)	Healthy controls (*N* = 54)	F	*P*
Sex (M/F)	25/24	17/19	26/28		
Age (years)	27.96 ± 9.70	28.89 ± 10.04	32.78 ± 10.01	3.385	0.037
Education (years)	12.55 ± 2.87	12.06 ± 4.08	13.67 ± 2.54	3.376	0.039
Age of illness onset (years)	26.59 ± 9.74	27.58 ± 10.09		0.292	0.649
Illness duration (months)	12.86 ± 14.13	9.89 ± 11.28		1.619	0.207
PANSS total score	71.67 ± 9.78	87.19 ± 12.79		1.309	< 0.001
P subscore	20.22 ± 4.42	25.97 ± 5.53		1.338	< 0.001
N subscore	17.59 ± 6.03	17.69 ± 6.96		1.231	0.942
G subscore	33.88 ± 5.94	43.44 ± 6.89		0.181	< 0.001
TNF-α levels (pg/mL)	87.51 ± 23.35	105.47 ± 36.21	75.25 ± 20.87	14.749	< 0.001
sTNF-α R1 levels (pg/mL)	3942.19 ± 938.49	3902.35 ± 1010.57	4399.69 ± 1254.91	2.821	0.063

PANSS: positive and negative syndrome scale; P: positive symptoms; N: negative symptoms; G: general psychopathological symptoms

Confirm that informed consent has been obtained from all subjects and/or their legal guardians.

### Clinical measures

The Structured Clinical Interview for DSM-IV Axis I Disorders-Patient Edition (SCID-I/P) was applied by two psychiatrists to screen the participants.The psychopathological symptoms were assessed using the Positive and Negative Syndrome Scale (PANSS) on the same day of blood sampling.Inter-rater concordance of assessments was over 0.8.The agitation symptoms were evaluated by the PANSS excitatory component (PANSS-EC), which included excitement (P4), hostility (P7), tension (G4), uncooperativeness (G8), and poor impulse control (G14). The PANSS-EC total score ≥ 14, with one or more items scoring ≥ 4, indicates the presence of agitation [7]. According to the criteria, the patients in this study were divided into non-agitated (*n* = 49) and agitated (*n* = 36) subgroups.

### TNF-α and sTNF-α R1 measurement

Plasma samples were collected using 5 ml of EDTAK2 disposable vacuum collection tubes (Beijing Dongfang Jianfeng Technology Co. Ltd) between 8 a.m. and 9 a.m., centrifuged (4 ℃, 3000 rpm, 10 min) (Thermo Fisher Scientific, America), aliquoted and stored at − 80 ℃. The plasma samples were collected to measure the TNF-α and sTNF-α R1 levels, which were measured using a sandwich enzyme linked immunosorbent assay (ELISA) kit from Beijing Rongxin Zhihe Biotechnology Co. Ltd., China. All samples were assayed by the same investigator, who was blinded to the clinical situation of the patients. The intra- and inter-assay variation coefficients were 4.67% and 9.72%, respectively.

### Data analysis

SPSS software version 22.0 (IBM, Armonk, NY, USA) and Graphpad prism 9.4 were used to make statistical analyses and graphs, respectively. One-way analysis of variance (ANOVA) with Tukey’s post-hoc tests was conducted on all the demographic data. When a significant difference between groups emerged for any variable in ANOVA, its effects on the plasma TNF-α and sTNF-α R1 levels were tested by adding these variables as covariates in a univariate analysis of covariance (ANCOVA) with Games-Howell’s post-hoc tests. Independent-sample t-tests were performed to analyze the differences between patients with schizophrenia in terms of their demographic and symptom variables. The correlation between TNF-α and sTNF-α R1 levels and clinical symptoms was performed using Pearson’s correlation analysis. We applied the Bonferroni correction to adjust for multiple tests. All tests were two-tailed, with a significance level of *p* < 0.05.

## Results

### Demographic and clinical data

The sex distribution was similar among the three groups. However, there was a significant difference in age and education years among the three groups (*p* = 0.037 and *p* = 0.039, respectively; Table 1). Post-hoc analysis showed that age and education differences in the patient subgroups were not significant. The subsequent statistical analyses were performed controlling for the effects of age and education level. The age at psychosis onset and illness duration were not significantly different between the patients with and without agitation (Table 1). However, the PANSS scores were significantly higher in patients with agitation than in those without agitation (*p* < 0.001).

### Plasma levels of TNF-α and sTNF-α R1

**Fig. 1:**
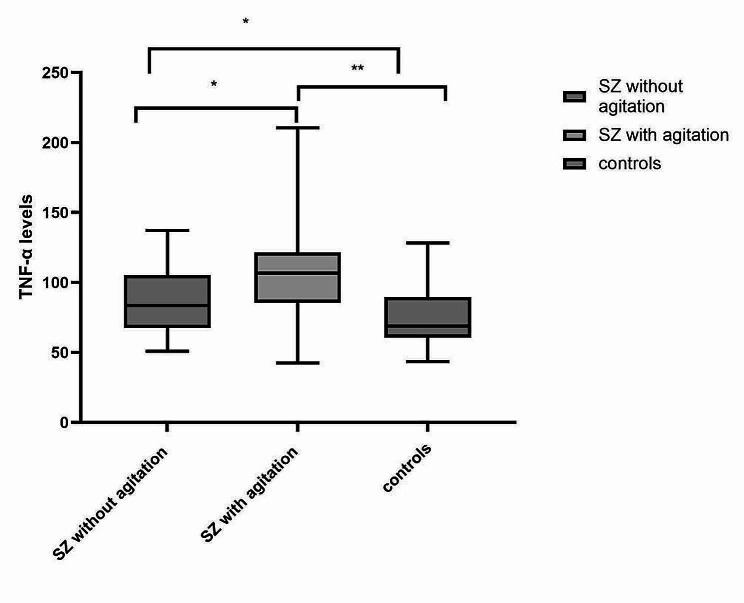
Plasma TNF-α levels among schizophrenics (SZ) without and with agitation and healthy controls (controls). ** *p* < 0.001, * *p* < 0.05)

The levels of TNF-α were significantly different among the FEPS with or without agitation, and the healthy control group (*F* = 14.749, *p* < 0.001; Table 1). The results of multiple comparisons with Games-Howell’s post hoc test showed that patients with schizophrenia with and without agitation had significantly different levels of TNF-α, and both were significantly different from the healthy controls after Bonferroni correction at *p* < 0.05/3 for three comparisons. The TNF-α level was highest in the agitated group and lowest in the control group (*p* < 0.05; Fig. 1). However, the sTNF-α R1 levels only approached a significant difference among the three groups (*p* = 0.063; Table 1).

### Correlation between symptoms and TNF-α levels

**Fig. 2:**
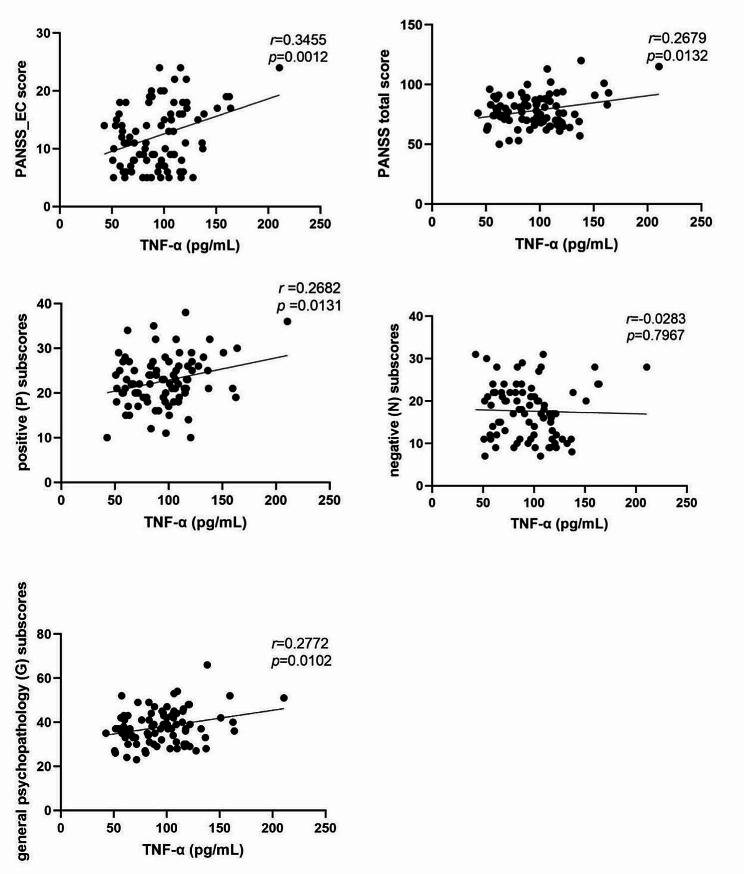
The relationships between the plasma TNF-α levels and the PANSS. EC: excitatory component

After controlling for age and years of education, the Pearson correlation analysis showed that the TNF-α level was positively correlated with the PANSS-EC score (*r* = 0.3455, *p* = 0.0012), PANSS total score (*r* = 0.2679, *p* = 0.0132), positive (P) (*r* = 0.2682, *p* = 0.0131) and general psychopathology (G) subscores (*r* = 0.2772, *p* = 0.0102) in all patients with schizophrenia (significant after Bonferroni correction at *p’s* < 0.05/3; Fig. 2). This finding indicates that the TNF-α level is related to the severity of the disease and may be involved in particular in the development of agitation symptoms.

### Correlation between symptoms and sTNF-α R1 levels

**Fig. 3:**
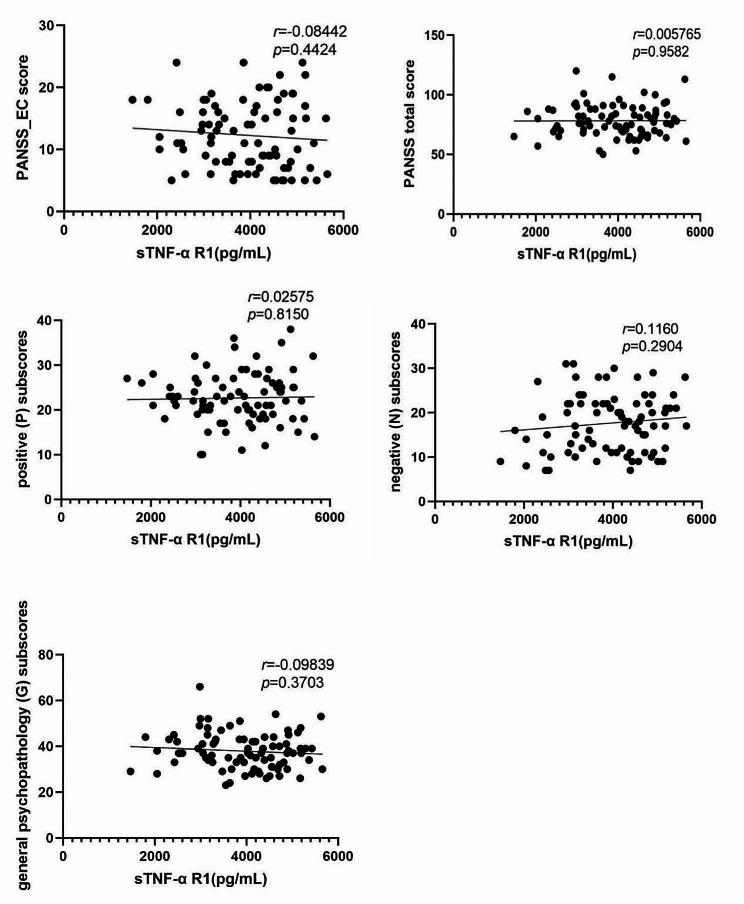
The relationships between the plasma TNF-α R1 levels and the PANSS-EC: excitatory component

After controlling for age and years of education, the Pearson correlation analysis showed that the sTNF-α R1 levels was not correlated with the PANSS_EC score, and subscores or total score of the PANSS in patient groups (*p’s* > 0.05; Fig. 3).

## Discussion

The main findings of this paper are as follows. The levels of TNF-α were significantly different among the FEPS with or without agitation, and the healthy control group.The TNF-α level was highest in the agitated group and lowest in the control group. However, the sTNF-α R1 levels only approached a significant difference among the three groups.After controlling for age and years of education, the Pearson correlation analysis showed that the TNF-α level was positively correlated with the PANSS-EC score, PANSS total score, positive (P) and general psychopathology (G) subscores in all patients with schizophrenia.The sTNF-α R1 levels was not correlated with the PANSS_EC score, and subscores or total score of the PANSS in patient groups.This finding indicates that the TNF-α level is related to the severity of the disease and may be involved in particular in the development of agitation symptoms.

A previous study found that TNF-α levels were higher in first-episode drug-naive (FEDN) patients with schizophrenia than those in healthy controls [12]. Another study reported that TNF-α levels were significantly higher in FEDN patients before treatment than those in chronic patients and healthy controls [8].Moreover, the FEPS reportedly had significantly higher TNF-α levels than healthy controls. After risperidone treatment, the TNF-α levels decreased significantly. No significant difference was found between the post-treatment cytokine levels in FEPS and healthy controls, suggesting that these alterations might be markers of FEPS [13]. Plasma TNF-α levels were significantly elevated in patients with early-onset schizophrenia (12–20 years old) compared with healthy subjects [14]. Our study examined first episode patients with schizophrenia (FEPS), and the findings that TNF-α levels were higher in these patients than those in healthy controls were consistent with previous researches. TNF-α levels were significantly lower in patients with chronic schizophrenia relative to healthy subjects [15]. This findings were inconsistent with our study, which might be related to the differences of patients, the previous study included patients with chronic schizophrenia, however, we recruited first episode patients. In the above studies, it has been shown that drug therapy can reduce the levels of TNF-α, so the difference between the above studies may be due to the selection of samples.

The TNF-α levels before treatment were significantly correlated to the PANSS scores in FEDN patients [8].Serum TNF-α levels were positively associated with negative symptoms, while no interaction between TNF-α correlated with any domains of the PANSS [12]. The correlation analysis showed a significant negative correlation between TNF-α levels and PANSS total scores [15].Our research have indicated that the TNF-α levels were positively correlated with the PANSS-EC score, PANSS total score, positive symptoms, and general psychopathological subscore.These inconsistent results might be due to multiple factors, including sample size, different episode history, illness duration, and antipsychotics. For the first time, we grouped agitation as an independent symptom and attempted to explore the correlation between cytokines and agitation symptoms.We found that levels of inflammatory cytokines are associated with the characteristic symptoms of schizophrenia.

Previous studies involving other mental diseases, such as depression and Alzheimer’s disease, had reported that the higher the agitation score of patients, the stronger the activity of HPA axis [16–18], suggesting the HPA axis participating in the correlation between TNF-α levels and agitation symptom in schizophrenia.Prior studies suggest that the TNF-α is associated with the activation of dopamine pathways, agitation symptoms are associated with an increased concentration of dopamine in the brain, and treatment is focused on blocking dopamine receptors and controlling agitation [19]. This may be another pathological mechanism by which TNF-α is associated with agitation symptoms.

A study founds that sTNF-α R1 levels were significantly elevated in patients with schizophrenia compared to healthy controls. In patients with schizophrenia, the levels of sTNF-α R1 was negatively correlated with global functioning [9]. A study that contradicts this result, sTNF-α R1 levels was lower in patients with schizophrenia. Serum sTNF-α R1 levels was negatively correlated with the negative subscale score and the total score of the PANSS [20]. Above results is in contrast with our findings of the difference in sTNF-α R1 levels between patients with schizophrenia and healthy controls only approached significance. And, after controlling for age and years of education, the Pearson correlation analysis showed that, the sTNF-α R1 levels was not correlated with the PANSS-EC score, PANSS total score, positive (P), negative(N) and general psychopathology (G) subscores in all patients with schizophrenia.These different results from our study may be due to sample factors, drug factors, as the above study did not limit the sample to patients with schizophrenia who were not first prescribed medication.

Based on previous research results, we enrolled to examine in-hospital patients, eliminating the influence of drugs, exercise, diet, and other aspects, and focus on the correlation between molecular factors and positive, negative, and agitation symptoms. This study has some limitations. First, the study was based on a cross-sectional design and could not investigate the direct causality between TNF-α and the development of agitation in patients with schizophrenia. Second, it is uncertain whether peripheral indicators of cytokines reflect similar changes in the central nervous system. Third, Many cytokines are involved in the immune response, and there is a strong correlation between different cytokines. The study of TNF-α and sTNF-α R1 may not be convincing enough, and multiple indicators can be selected according to different mechanisms in subsequent studies. Meanwhile, the absence of controlling for the effects of antipsychotic use is one of the limitations of this study. All patients were prescribed antipsychotic medications without delay upon admission, which may affect inflammatory cytokine levels. However, the usage duration of all patients was less than two weeks and was not enough to exert the therapeutic effect [10, 11]. Moreover, there is no observed correlation between CPZ and TNF-α level(*p* = 0.855), suggesting that the impact of antipsychotic medications on our findings was likely minimal. Therefore, our present findings remain preliminary.

In conclusion, altered peripheral TNF-α levels are associated with the psychopathology, and in particular with agitation in patients with schizophrenia.

## Data Availability

The data that support the findings of this study are available from Beijing Huilongguan Hospital Research, but restrictions apply to the availability of these data, which were used under license for the current study and so are not publicly available. The data are, however, available from the authors upon reasonable request and with the permission of the Beijing Hui long guan Hospital Research. Email: yltan21@126.com.
